# Identification of the palliative phase in people with dementia: a variety of opinions between healthcare professionals

**DOI:** 10.1186/s12904-015-0053-8

**Published:** 2015-11-04

**Authors:** Jasper van Riet Paap, Elena Mariani, Rabih Chattat, Raymond Koopmans, Hélène Kerhervé, Wojciech Leppert, Maria Forycka, Lukas Radbruch, Birgit Jaspers, Kris Vissers, Myrra Vernooij-Dassen, Yvonne Engels, on behalf of the IMPACT research team

**Affiliations:** Scientific Institute for Quality of Healthcare (IQ healthcare), Radboud university medical center, P.O. Box 9101, 6500 HB Nijmegen, The Netherlands; Department of Psychology, University of Bologna, Viale Berti Pichat 5, 40127 Bologna, Italy; Department of Primary and Community Care, Radboud university medical center, P.O. 6500 HB Nijmegen, The Netherlands; Radboud Alzheimer Centre, Radboud university medical center, P.O. Box 9101, 6500 HB Nijmegen, The Netherlands; Joachim en Anna, Centre for specialized geraitric care, Nijmegen, The Netherlands; Department of Geriatrics, Broca Hospital, AP-HP, 54-56 rue Pascal, 75013 Paris, France; Department of Palliative Medicine, Poznan University of Medical Sciences, 61-245, Poznan, Poland; Department of Palliative Medicine, Universitätsklinikum Bonn, Sigmund-Freud-Street 25, 53127 Bonn, Germany; Department of Palliative Care, Malteser Hospital Bonn /Rhein-Sieg, Bonn, Germany; Department of Anaesthesiology, Pain and Palliative Medicine, Radboud university medical center, P.O. Box 9101, 6500 HB Nijmegen, The Netherlands; Kalorama Foundation, Nijmegen, The Netherlands

**Keywords:** Palliative care, Dementia, Long-term care, Nursing home, Staff views, Europe

## Abstract

**Background:**

People with dementia can benefit from a palliative care approach. Recommendations, such as those of the EAPC have been proposed to strengthen the provision of palliative care for this group of patients. Yet, it remains challenging for professionals to identify when a person with dementia is in need of palliative care. The objective of this study therefore was to explore when professionals in long-term care settings consider a person with dementia in need of palliative care.

**Methods:**

Teams with in total 84 professionals working in 13 long-term care settings from 6 countries (France, Germany, Italy, Norway, Poland and the Netherlands) received a case-vignette concerning a person with dementia recently admitted to a nursing home. Teams were asked to discuss when they considered people with dementia eligible for palliative care. The constant comparative method was used to analyse their answers.

**Results:**

Three different time points in the disease trajectory when people with dementia were considered to be eligible for palliative care were extracted: (1) early in the disease trajectory; (2) when signs and symptoms of advanced dementia are present; and (3) from the time point that curative treatment of co-morbidities is futile. Yet, none of these time points was uniformly considered by the professional teams across Europe. In some cases, professionals working in the same nursing home didn’t even reach consensus when considering persons with dementia eligible for palliative care.

**Conclusion:**

The results of the study identified that professionals across Europe have different opinions regarding the time point when to consider a person with dementia in need of palliative care.

## Background

Worldwide, about 36 million persons have dementia [1]. People with advanced stages of dementia have complex physical and psychological needs [2, 3]. Many suffer from symptoms such as pain, agitation, dyspnea, neuropsychiatric symptoms and depression [4], which threatens the quality of their lives as well as that of their relatives. Appropriate palliative care can deal with the needs and preferences of people with dementia and their families [2]. However, access to palliative care services for people with dementia is less defined than for patients with cancer [5]. Professionals in dementia care often lack the necessary skills to anticipate the changing palliative care needs of a person with dementia [5–7]. Therefore, people with dementia are more frequently hospitalized and too often receive burdensome interventions [8]. Moreover, compared to patients with other life-threatening diseases, they are less likely to receive advance care planning [6], are less frequently referred to palliative care teams or hospice care [6] and more often experience symptoms for a longer period of time [9].

Dementia is more and more acknowledged as a life-threatening disease [5]. Time from diagnosis until death varies from two to 20 years [2, 10]. This protracted course of dementia makes it difficult for persons with dementia and their families, as well as for professionals to discuss end-of-life issues, such as advance treatment decisions, preferred place of care and death or lasting power of attorney [2, 4, 5]. Consequently, people with dementia are often not involved in discussions about preferences and needs early in the disease [11], when their cognitive impairment does not yet impede their participation in the decision-making process.

Recently, the European Association for Palliative Care published a white paper on defining palliative care in dementia [12]. One of the recommendations is to consider the time point of the diagnosis of dementia as the starting point of palliative care [12]. However, there is still an ongoing discussion on the identification of the palliative phase in dementia. Besides, people with dementia have unequal access to palliative care services compared to patients with cancer [13]. Therefore, the aim of this study was to explore when professionals in long-term care (LTC) facilities across Europe consider a person with dementia in need of palliative care.

## Methods

The EU-funded Seventh Framework IMPACT project (**IM**plementation of quality indicators in **PA**lliative **C**are s**T**udy) aims to develop and tailor national and setting-specific strategies to improve the organisation of palliative care in several European countries. As part of this study, a pre-post test was conducted in 40 services across Europe to assess the organisation of palliative care of long-term care settings, in which also a case-vignette was used. Case-vignettes have been used in a variety of settings [14–18], and they offer a promising alternative for the assessment of the performance of healthcare professionals. Case-vignettes consist of ‘text, images or other stimuli to which research participants are asked to respond’ [16]. In this study, the case-vignette was created in a way that it explicitly excluded clinical details of the depicted subject (e.g. about the prognosis, symptoms, etc.) in order to stimulate discussion. The present paper presents the results of the case-vignette about identifying the palliative phase in people with dementia.

### Case-vignette

Specific characteristics of a person with dementia were drafted by a general practitioner (Professor of Primary Care for Older People, SI), and used to develop a case-vignette in English. The case-vignette was presented to the IMPACT project team (consisting of 14 clinicians and researchers). After having fine-tuned the concept case-vignette with their feedback (see Table 1), the English case-vignette was translated into the local languages of the participating countries involved in the project, using a forward-backwards translation. Subsequently, researchers were asked to pilot test the translated case-vignette with at least two professionals in their country. These professionals were asked to evaluate the comprehensiveness and clarity of the vignettes.

**Table 1:** Case-vignette of a person with dementia

Mrs. White is 83 years old. She has been married for 56 years to Charles. They have one child, Lucy, who is 47, and who keeps in regular contact with them.
Mrs. White was diagnosed with dementia about 9 years ago. Until recently, she lived with her husband in a house in the country. Because Mrs. White can get quite aggressive when she does not understand what is going on, her husband can no longer deal with her at home. Therefore Mrs. White recently moved to a nursing home.
Question: Please explain if and when you would consider Mrs. White as a person in need of palliative care?

### Setting and participants

At least two LTC settings for people with dementia were purposefully selected per country. These LTC settings had to have at least one year of experience in the provision of palliative care. Each of the selected LTC settings recruited members from their multidisciplinary team (Table 2). Selection criteria for these team members were being involved in direct patient care or, at least, having knowledge of direct patient care. In each setting, one professional was appointed by the researchers as contact person.

**Table 2:** Participating professionals per nursing home

	DE-1	DE-2	FR-1	FR-2	IT-1	IT-2	IT-3	NO-1	NO-2	NL-1	NL-2	PL-1	PL-2
Physician	-	-	1	1	1	1	1	-	-	-	-	1	1
Nurse	3	2	1	1	1	1	1	2	6	3	5	2	3
Healthcare assistant	4	2	1	3	1	1	-	3	1	3	-	-	-
Psychologist	-	-	-	1	1	2	-	-	-	-	-	1	1
Social worker	-	-	-	-	-	-	-	-	-	-	-	1	-
Other	-	-	2	5	3	2	4	-	-	1	-	1	3
Total	7	4	5	11	7	7	6	5	7	6	5	6	8

*DE* Germany, *FR* France, *IT* Italy, *NO* Norway, *PL* Poland, *NL* the Netherlands

### Data collection

The multidisciplinary teams participated in a meeting in which the case vignette was presented. In each setting, the contact person chaired this meeting. This person was instructed about the purpose of the meeting. The participants did not receive a definition of palliative care as this would have biased the results. In this study, participants were stimulated to share their own definitions and clinical perceptions about palliative care. Participants were also instructed to consider the depicted person as one of their own residents and were asked: *‘Please, could you explain if and when you would consider Mrs. White as a person in need of palliative care?’.* Instructions also stated that consensus within the multidisciplinary team was not important; different opinions could exist. Within each multidisciplinary group, the chair person summarized and documented the answer (s) according to a predefined template divided into three main sections: job titles of participants; outcomes of the discussion; observational analysis of the discussion process. The chair person was asked to translate the answers into English and to provide detailed information about the process how they came to their answers (e.g. specifying if there was immediate consensus, whether there was a long discussion, if requests of clarifications occurred and reactions of the participants). Subsequently, the chair persons submitted their answer (s) as open text into an online data-registration tool (a web-based data registration tool based on LimeSurvey). If any of the information was unclear, the chair person was contacted to provide further explanations.

### Analysis

In each non-English country, the researcher translated the answers of the vignette into English. Two researchers (JvRP and EM) independently coded the data by using a constant comparative method [19]. First, each researcher conducted the comparison within single interviews, developing and labeling categories with appropriate codes in order to outline the core concepts of the interviews. Second, a comparison between interviews was conducted, combining the codes in clusters, in order to define the concepts and identify similarities and differences between interviews [20]. The two researchers discussed their codings until consensus was reached. Regular contact (face-to-face, by Skype and by email) was used during the analysis to refine codes and to group the codes into unique categories. When no consensus could be reached, a third researcher was consulted (YE). Themes and categories were regularly fed back and discussed with two other authors (MVD and YE).

### Ethical considerations

The Medical Ethics Committee of the district Arnhem-Nijmegen has declared that this study doesn’t fall within the remit of the Medical Research Involving Human Subjects Act (WMO) (registration number 2012/075). This means that this study can be carried out without an approval by an accredited medical ethics committee.

## Results

Thirteen nursing homes in six European countries (France, Germany, Italy, Norway, Poland and the Netherlands) participated in the vignette study. In all nursing homes, the staff were responsible for the provision of palliative care. In Germany, Poland and one Dutch nursing home, staff had 24/7 accessibility to specialist services, whereas in the other nursing homes this fluctuated between working hours only to none at all. In one German, the Italian and Dutch nursing homes, an end-of-life care pathway was commonly used for the last three days of life of a person in need of palliative care.

In total, 84 professionals took part in the multidisciplinary team discussions (Table 2). Professionals in nine nursing homes considered Mrs. White in need of palliative care (Table 3). In four nursing homes, professionals stated that Mrs. White was not in need of palliative care. The multidisciplinary team reached consensus on their view when to consider Mrs. White in need of palliative care in ten nursing homes. The opinions of the multidisciplinary teams varied so much in the remaining three nursing homes, that they were not able to reach consensus during the discussion of the vignette.

The reasons why the multidisciplinary teams did or did not consider Mrs. White in need of palliative care varied and could be grouped into three categories representing different attitudes of staff members on the entry point for palliative care: (1) palliative care starts early in the disease trajectory, (2) palliative care starts when signs and symptoms of advanced dementia are present, and (3) palliative care starts when curative treatment for co-morbidities is no longer possible.

### Palliative care should start early in the disease trajectory

Professionals in a German nursing home (DE-2) unambiguously agreed that Mrs. White was a person in need of palliative care from the day she moved in. A similar answer came from an Italian nursing home (IT-2), whereby some professionals stated that dementia is a terminal disease and consequently all their residents, including Mrs. White, should be treated as people in need of palliative care. In the Netherlands, the vignette generated a debate between professionals in a nursing home (NL-1): participating health care assistants first had the impression that palliative care only involved the last three days of life. Two nurses, however, persuaded the health care assistants that they should consider people like Mrs. White, with a diagnosis of dementia, as in need of palliative care. In the end, the multidisciplinary team agreed that Mrs. White was in need of palliative care. Similarly, some professionals of a Norwegian (NO-2) and of a Polish (PL-2) nursing home argued that people with early-stage dementia should be considered in need of palliative care.

### Palliative care should start when clinical symptoms of advanced dementia are present

A German nursing home (DE-1) used a self-developed assessment tool to identify palliative care needs and symptoms of their own residents. For that reason, the members of this team agreed that if Mrs. White would meet the criteria of this assessment tool, they would consider her in need of palliative care. Similarly, staff in an Italian nursing home (IT-1) unanimously considered Mrs. White in need of palliative care if she suffered from serious communication deficits, physical disorders, pain and severe agitation. Yet, in a second Italian nursing home (IT-2), staff were not able to reach consensus whether to consider Mrs. White in need of palliative care. Some professionals did mention that palliative care is exclusively applicable for people with advanced dementia. In a third Italian nursing home (IT-3), team members agreed that a person with advanced dementia would be considered in need of palliative care. However, they considered that Mrs. White, as depicted in the vignette, did not show symptoms of advanced dementia. An analogous concept was expressed by professionals in two French nursing homes (FR-1 and FR-2). They unanimously agreed that Mrs. White was not in an advanced stage of dementia and therefore not in need of palliative care. Professionals in a Norwegian nursing home (NO-1) also shared this opinion. However, in another Norwegian nursing home (NO-2), staff were not able to reach consensus. Some stated that palliative care is applicable for people with dementia with a short life expectancy. Lastly, Polish professionals (PL-1 and PL-2) referred to the time point in the disease trajectory in which dementia symptoms seriously hamper a person’s autonomy and demand intensive medical and nursing care.

### Palliative care should start when curative treatment for co-morbidities has no longer a beneficial effect

Professionals from a Dutch nursing home (NL-2) agreed that at the time Mrs. White is experiencing physical diseases and the doctors decide not to treat these anymore, she should be considered in need of palliative care. In a Norwegian nursing home (NO-2) professionals did not reach consensus, and only some of them considered Mrs. White in need of palliative care when she would no longer benefit from medical or surgical treatment.

**Table 3:** Professionals’ consideration if and when a person with dementia is in need of palliative care

	*DE-1*	*DE-2*	*FR-1*	*FR-2*	*IT-1*	*IT-2*	*IT-3*	*NO-1*	*NO-2* ^*a*^	*NL-1*	*NL-2*	*PL-1*	*PL-2* ^*a*^
Do you consider the person in the case vignette to be in need of palliative care?	Yes	Yes	No	No	Yes	Yes	No	No	Yes	Yes	Yes	Yes	Yes
When she is in the early stage of dementia	-	+	-	-	-	+	-	-	+	+	-	-	+
When she has signs and symptoms of advanced dementia	+	-	+	+	+	+	+	+	+	-	-	+	+
When she has no more beneficial effect of curative treatment for co-morbidities	-	-	-	-	-	-	-	-	+	-	+	-	-

*DE* Germany, *FR* France, *IT* Italy, *NO* Norway, *PL* Poland, *NL* The Netherlands

^a^Services where there was no consensus between professionals

## Discussion

This study highlights the challenges faced by professionals working in long-term care settings with people with advanced dementia in defining the time point when palliative care should start. With the help of a case-vignette, we identified three time points in the disease trajectory of a person with dementia that teams of nursing home professionals considered as the moment to start palliative care: (1) from the early stages of dementia, (2) when signs and symptoms of advanced dementia are present, and (3) from the time point that curative treatment for co-morbidities is futile. Discrepancies were found not only between European countries, but also between staff members working in the same LTC setting. In some nursing homes, for example, professionals disagreed about the time point a person with dementia is in need of palliative care. Also between countries, different time points when a person becomes eligible for palliative care were mentioned.

However, most professionals described that palliative care should be provided when a person with dementia shows symptoms indicating the advanced stage of dementia is approaching the end-of-life phase, such as swallowing disorders, pain, or when the body does not respond to food or liquids anymore.

Birch et al described that professionals often find it difficult to recognize unmet palliative care needs of people with dementia because the progression of dementia differs in each person [2]. The progression towards the advanced stages of dementia, for example, remains unpredictable [2, 10]. Prognostic indicators to identify end-stage dementia may increase the availability of palliative care options for people with dementia and their families [8], but they are often used too late and seem unreliable to predict a person’s death [21]. Some of the professional teams in our study responded that the early stages of dementia can be considered as the time point palliative care starts. Black et al [22] described that recognizing the needs and preferences of people with dementia early in the disease trajectory facilitates the involvement in the decision-making process and advance care planning.

Professionals in two nursing homes considered the time point that curative treatment for co-morbidities is futile and does not improve the person’s quality of life as the starting point for palliative care. However, similarly to using prognostic indicators, considering the time point when co-morbidities cannot be treated might be too late in the disease trajectory to provide proactive palliative care as the cognitive abilities of a person with dementia have declined too much so that the person is no longer able to participate in the decision making process and advance care planning.

Before group discussion, some professionals even considered the last days of life as the starting point for palliative care, meaning when the patient is about to die. Although we only reported about the final considerations of the professionals, it is important to note that this could potentially be a fourth time-point.

Differences between countries in identifying the time point of the palliative phase were expected, because of different cultures and national regulations for palliative care [23]. However, even within countries, different opinions about the time point of the palliative phase were identified. Thereby, there appeared to be differences in definition about palliative care between services. Although important consensus statement reports such as the EAPC’s White Paper, defining optimal palliative care in older people with dementia [12], have been developed and dissiminated, they are not sufficient to overcome these barriers. Access to palliative care therefore depends on the perceptions of palliative care professionals about when palliative care becomes appropriate for people with dementia. There is a need for further research into the differences palliative care makes to quality of life and end of life care for people with dementia, and the perceptions of palliative care professionals about the value of engaging in the care of people with dementia. Knowledge about and experience in palliative care of professionals working in dementia care therefore need to be improved [24]. Teaching professionals to lead their caregiving by needs probably might be the most important step in providing timely palliative care in each phase of the disease. Reaching consensus about the definition of palliative care and subsequently about the time point of the palliative phase is therefore necessary [25].

This study can contribute to the ongoing discussion on this topic by showing that there are three time points when nursing home professionals consider a person with dementia in need of palliative care: early in the disease trajectory, advanced dementia or when curative treatment for co-morbidities has no more beneficial effect. Even within services, it appeared that sometimes there were different opinions regarding the starting point of the palliative phase. Future attempts to define the optimal time point of the palliative phase in dementia should acknowledge these differences.

### Strengths and limitations

This study contributes to our knowledge about the challenges that professionals working in LTC settings experience during their daily work with people suffering from dementia, particularly regarding their palliative care needs. Besides, it is the first study in which we get insights in how professionals that work with people with dementia on a daily basis define the starting point of palliative care. This is an important addition to the theoretical studies on this topic [12, 26]. However, some limitations have to be taken into account. Participants did not receive a definition of palliative care prior to their discussion about the starting point of palliative care as it was the aim of this study to allow them to share their own definitions and clinical perceptions about palliative care. Their opinions may have therefore been influenced by the type of palliative care intervention available in their service. If we would have provided the WHO definition of palliative care, their own meaning would probably have been influenced by this.

Second, as answers provided by the participants were anonymized, it was not possible to identify differences in the personal perspectives of the healthcare professionals. If these would have been reported, the anonymity of the participants would have been at stake because of the few services and professionals involved.

Third, although data was collected in nursing homes in six European countries, the data may not be representative for all services at the regional or national healthcare system in the respective countries. This study was too small to grasp such differences. Further multicenter and multinational studies have to be conducted to analyze regional or national differences regarding the starting point of the palliative phase.

Fifth, it was the aim of this study to explore when professionals in long-term care settings consider a person with dementia in need of palliative care and not to reach consensus about the time point that the palliative phase starts in people with dementia. Future research can therefore use the three time points identified in this paper, to further explore the possibilities of reaching consensus about the time point of the palliative phase in people with dementia within and between services.

## Conclusion

The findings from this study show that professionals across Europe have different opinions regarding the time point when to consider a person with dementia in need of palliative care. The range of opinions described in this study lead to the recommendation that multiple methods for information and education of staff members should be pursued to improve palliative care policy and service delivery for people with dementia.
